# Core needle biopsy of periacetabular chondrosarcoma often results in undergrading but does not change management by experienced orthopaedic oncologists

**DOI:** 10.1038/s41598-025-93246-4

**Published:** 2025-09-30

**Authors:** Hayden Harvard, Megan A. Badejo, Nicole Levine, Nicole Cantor, Julia Visgauss, Brian Brigman, William C. Eward

**Affiliations:** 1 Dept. of Orthopaedic Surgery, Duke University Hospital, Durham, NC USA; 2 Duke Cancer Institute, Duke University Hospital, Durham, NC USA; 3 Mercer University School of Medicine, Savannah, GA USA; 4 Campbell University School of Osteopathic Medicine, Lillington, NC USA

**Keywords:** Chondrosarcoma, Histology, Pathology, Periacetabular, Sarcoma, Tumour heterogeneity, Oncology, Surgical oncology

## Abstract

There is discrepancy in the histologic concordance of pre-operative biopsy grading and post-surgical resection grading of periacetabular chondrosarcoma. This discrepancy poses a question as to whether the chosen treatment should depend upon on the initial assessment of the tumor grade. This is a retrospective review of patients with periacetabular cartilaginous tumors who were treated between the years 2000 and 2022 at a single institution. All patients included in the analysis underwent surgical resection. The pre-operative biopsy and post-surgical resection histological grades were compared. Concordance and treatment implications were evaluated. Twenty-two pre-operative Image Guided Core Needle Biopsies (IGCNB) were performed. There was concordance between pre-operative and surgical resection histological grade in only 9 cases, giving a (9/22) 40.9% accuracy rate. In total there were 13 cases of discrepancy between pre-operative biopsy and surgical resection regarding histological grade. In 12 cases of discrepancy, surgical resection histopathology revealed a higher grade than that of pre-operative biopsy histopathology. In one case of discrepancy, pre-operative IGCNB biopsy histology revealed a high-grade chondrosarcoma while the surgical resection histology revealed an intermediate-grade. There were no situations in which the plan made based on a preoperative biopsy resulted in an inappropriate treatment decision, even when there was not concordance between preoperative diagnosis and diagnosis after definitive resection. Significant discrepancy in grading was observed with pre-operative IGCNB of periacetabular cartilaginous lesions. However, while 12 out of the 13 discordant cases (92.3%) underestimated the true grade of the tumor, there was no impact on appropriateness of the selected treatment, suggesting that accuracy of grade is not an important factor to consider in this location.

**Supplementary Information:**

The online version contains supplementary material available at 10.1038/s41598-025-93246-4.

## Background

Chondrosarcoma is a malignant hyaline cartilage neoplasm most commonly diagnosed in adults older than 50 years of age and typically affects the long bones and the pelvis^1,2^. It represents the most common primary bone tumor affecting the periacetabular region, followed by osteosarcoma and Ewing’s sarcoma^3^. Periacetabular tumors are unique in that curative treatment with wide resection is associated with significant patient morbidity. Given the challenges associated with obtaining an open biopsy in this region, percutaneous biopsy methods have become a favorable means to obtain a tissue sample. However, percutaneous biopsy of pelvic chondrosarcoma results in a relatively small yield of diagnostic tissue. Given the inherent challenges with diagnosis and grading of chondrosarcoma, this increases the risk of sampling bias and underestimation of tumor grade^4–6^. Therefore, management of these patients is often determined through a collective group of interdisciplinary physicians by carefully weighing the clinical presentation and imaging findings and incorporating these with the histopathological results. This differs from the majority of oncologic surgeries for primary bone cancers where treatment does not begin until the histologic diagnosis and grade are confirmed, even if this requires an open biopsy for lesions where the imaging suggests a different diagnosis or a different grade than the tissue sampled percutaneously. While advances in our understanding of the genetic makeup and biological nature of chondrosarcoma are being made, we still have yet to translate these findings into therapeutic treatments^7^. Therefore an additional aspect of managing chondrosarcoma is that while a variety of other treatments (radiotherapy, chemotherapy, hormonal therapy, immunotherapy) have been used to treat other sarcomas^8,9^, these have very limited value in conventional chondrosarcoma as most subtypes are treated with surgery alone (with exception of the mesenchymal and dedifferentiated subtypes which may be treated with adjuvant/neoadjuvant chemotherapy). Due to the morbidity that attends open biopsy of the periacetabular region, decisions about periacetabular chondrosarcoma are often made based on a percutaneous needle biopsy even when there is diagnostic uncertainty about the grade or subtype.

To better evaluate the accuracy between pre- and post-operative histopathology, we performed a retrospective review of patients with periacetabular cartilaginous tumors at a single institution. The current protocol at our institution regarding suspected chondrosarcoma of the periacetabular region is as follows: Image guided preoperative biopsy is first attempted by a musculoskeletal radiologist, preferably with a core needle biopsy. Planning of the biopsy path is discussed with an orthopaedic oncologist. If the preoperative biopsy reveals a cartilaginous tumor of any grade and the patient’s clinical presentation, imaging, and histological findings are suggestive of chondrosarcoma, as decided on by the multidisciplinary team, then wide resection of the lesion is typically recommended. Open biopsy is typically not pursued to obtain definitive grading of periacetabular lesions, given the prognosis and biological behavior of cartilage producing tumors in the pelvis. However, this raises the question of whether we are too aggressive with surgery for patients who may have a low-grade periacetabular cartilaginous tumor on initial biopsy. To answer this question, we decided to evaluate the concordance of tissue diagnosis and histologic grade between preoperative and postoperative tissue samples. Furthermore, we aimed to determine whether any changes in diagnosis or grading following resection and complete histopathologic review of the entire lesion would have resulted in any deviations from the executed surgical plan. This represents the first such investigation in this topic which was highlighted as an area of controversy at the recent Birmingham Orthopaedic Oncology Meeting^10^.

## Methods

Following approval by our Institutional Review Board, we conducted a retrospective chart review of patients with periacetabular cartilaginous tumors of bone who were treated between the years 2000 and 2022 at our institution (662 patients). We further narrowed to 507 patients who had undergone excision or radical resection of tumor in soft tissue or bone about the pelvis, and 155 pathology reports were identified using the keywords “chondroid”, “cartilage”, “chondrosarcoma” in conjunction with “pelvis”, “ilium”, “acetabulum”, “pubis”, “sacrum” and “ischium”) using our institutions database. After accounting for duplicates, we excluded all patients with a preoperative diagnosis other than chondroid lesion, cartilage lesion, chondroblastoma, chondromyxoid fibroma, and chondrosarcoma, and all lesions located outside of the pelvis. We further excluded all patients without periacetabular involvement, patients with pre-operative diagnosis consistent with chondroblastoma or chondromyxoid fibroma, and those with incomplete medical records that would not allow us to compare biopsy results. As a result, 22 patients were identified with a diagnosis of chondroid lesion, cartilage lesion, or chondrosarcoma and included in our final analysis.

All patients underwent preoperative biopsy using image guided core needle biopsy (IGCNB). The image guided core needle biopsies were performed under either ultrasonography or CT guidance. A total of 22 IGCNB were performed. For the 14 biopsies done at our institution, the method and approach were chosen and decided upon by the treating orthopaedic surgeon and musculoskeletal radiologist following interdisciplinary discussion. Of the 8 biopsies performed at outside institutions, six patients’ tissue were able to be retrieved, reviewed, and graded by our institution’s musculoskeletal pathologists. Patients with preoperative biopsy consistent with cartilaginous periacetabular tumor and with high grade imaging and clinical features (such as cortical erosion, soft tissue extension, periosteal reaction, pain, etc.) suggestive of chondrosarcoma were managed with internal or external hemipelvectomy. For patients who underwent internal hemipelvectomy reconstruction was performed if desired by the patient and mechanically feasible.

Each patient’s preoperative and surgical pathology reports were reviewed to identify the histological grade and diagnosis. To standardize the nomenclature found in pathology reports, when the term Grade 1 was reported it was recoded as “low-grade”, Grade 2 as “intermediate grade”, Grade 3 as “high-grade”, and dedifferentiated was retained. When a histology report noted the presence of mixed grade tumor the higher grade was documented for comparison in our analysis. The histological grade based on preoperative biopsy and surgical specimen was then compared to identify the concordance. Our primary outcome was overall concordance between pre- and post-operative histologic grade.

## Results

A total of 22 patients, with a mean age at presentation of 55.7 years of age (range, 30–72 years), and a male-to-female ratio of 14:8, were analyzed in this retrospective chart review. All 22 preoperative biopsies performed were done by means of Image Guided Core Needle Biopsy (IGCNB) (Table 1).

**Table 1:** Concordance of histological grading between pre-operative biopsy and surgical resection pathology.

	Total Number	Concordant	Discrepant
Image Guided Core Needle Biopsy	22	40.9% (9/22)	59.1% (13/22)

Of the 22 included patients, 8 patients were assigned a diagnosis of low-grade cartilaginous tumor, 11 patients received a diagnosis of intermediate-grade chondrosarcoma, two a diagnosis of high-grade chondrosarcoma, and one a diagnosis of dedifferentiated chondrosarcoma (Table 2) based on initial biopsy.

**Table 2:** Summary of all 22 cases of periacetabular chondrosarcomas.

Patient	Sex	Age	Location of tumor	Biopsy method	Pre-operative biopsy	Surgical resection Pathology	Hemipelvectomy Subtype	Final Diagnosis
10	M	66	Acetabulum	IGCNB	Low-grade	Intermediate-grade	I/II/III	Chondrosarcoma
17	F	43	Acetabulum	IGCNB	Low-grade	Intermediate-grade	I/II/III	Chondrosarcoma
18	F	64	Acetabulum	IGCNB	Low-grade	Intermediate-grade	II	Chondrosarcoma
19	M	56	Acetabulum/ilium	IGCNB	Low-grade	Intermediate-grade	I/II	Chondrosarcoma
21	M	30	Acetabulum/pubis	IGCNB	Low-grade	Intermediate-grade	I/II/III	Chondrosarcoma
25	M	65	Acetabulum/ischium	IGCNB	Low-grade	Intermediate-grade	II/III	Chondrosarcoma
38	F	64	Acetabulum	IGCNB	Low-grade	Intermediate-grade	II	Chondrosarcoma
35	M	68	Acetabulum/ischium	IGCNB	Low-grade	Dedifferentiated	I/II/III/IV External	Dedifferentiated Chondrosarcoma
8	M	35	Acetabulum/pubis	IGCNB	Intermediate-grade	Intermediate-grade	II/III	Chondrosarcoma
12	M	64	Acetabulum/ilium	IGCNB	Intermediate-grade	Intermediate-grade	I/II/III/IV	Chondrosarcoma
22	M	52	Acetabulum/ilium	IGCNB	Intermediate-grade	Intermediate-grade	I/II	Chondrosarcoma
24	M	53	Acetabulum/pubis	IGCNB	Intermediate-grade	Intermediate-grade	II/III	Chondrosarcoma
26	M	55	Acetabulum/ilium	IGCNB	Intermediate-grade	Intermediate-grade	I/II/III	Chondrosarcoma
27	F	48	Acetabulum/pubis	IGCNB	Intermediate-grade	Intermediate-grade	II/III	Chondrosarcoma
28	F	72	Acetabulum/pubis/ilium	IGCNB	Intermediate-grade	Intermediate-grade	I/II/III/IV	Chondrosarcoma
36	M	65	Acetabulum/ilium/ischium/thigh	IGCNB	Intermediate-grade	Intermediate-grade	I/II/III/IV External	Chondrosarcoma
5	M	66	Acetabulum/pubic root	IGCNB	Intermediate-grade	High-grade	I/II/III	Chondrosarcoma
14	F	56	Acetabulum	IGCNB	Intermediate-grade	High-grade	I/II/III	Chondrosarcoma
16	M	49	Acetabulum/ilium	IGCNB	Intermediate-grade	High-grade	I/II/III	Chondrosarcoma
23	M	56	Acetabulum/ilium	IGCNB	High-grade	Intermediate-grade	I/II/III/IV	Chondrosarcoma
13	F	61	Acetabulum/pubis	IGCNB	High-grade	Dedifferentiated	II/III	Chondrosarcoma
34	F	39	Acetabulum	IGCNB	Dedifferentiated	Dedifferentiated	II	Dedifferentiated Chondrosarcoma

In each of the 8 patients with a low-grade cartilaginous tumor, clinical and imaging features were suggestive of chondrosarcoma and hemipelvectomy was recommended. Of this subgroup, final diagnosis following hemipelvectomy was intermediate-grade chondrosarcoma for 7 patients and dedifferentiated chondrosarcoma for 1 patient (Table 2).

In the 11 patients with a pre-operative diagnosis of intermediate-grade chondrosarcoma, the final diagnosis following hemipelvectomy remained unchanged for 8 patients and for 3 patients was recategorized as high-grade chondrosarcoma. Two patients received a pre-operative diagnosis of high-grade chondrosarcoma and one a diagnosis of dedifferentiated chondrosarcoma. One of the high-grade chondrosarcoma patients was downgraded to intermediate-grade pathology, while the other was re-classified as dedifferentiated on final pathology. The patient with dedifferentiated chondrosarcoma on initial biopsy had concordant pre- and post-resection histology (Table 2).

In evaluating the entire cohort, there was agreement between pre-operative and surgical resection histological grade in 9 cases using IGCNB, giving a (9/22) 40.9% accuracy rate overall. In total there were 13 cases of discrepancy (59.1%) between pre-operative biopsy and surgical resection histological grade, with all but one case resulting in upgrading on final pathology (Table 1). The only tumor that was downgraded went from high-grade to intermediate-grade, and it should be noted that in chondrosarcoma “intermediate grade” tumors are still considered to have aggressive behavior and are treated the same as “high-grade” tumors.

## Discussion

Chondrosarcomas represent a diverse and challenging group of tumors that present a particular challenge to the orthopaedic oncologist. First, accurate classification and grading of cartilaginous tumors is inherently subjective, often ambiguous, and disproportionately affected by sampling bias compared to other malignancies. Given that they are often resistant to chemotherapy and radiation, curative treatment almost always requires wide surgical resection^1,11,12^. Chondrosarcomas of the axial skeleton represent an especially challenging area to obtain a preoperative biopsy and diagnosis. Preoperative biopsy is therefore often performed by means of percutaneous image guided techniques. One challenge of these biopsy techniques is the limited amount of tissue that can be obtained for review, in a tumor that is inherently at risk of sampling error. For many patients with chondroid lesions about the pelvis who undergo percutaneous biopsy, the histologic diagnosis may be left with ambiguity about grade or subtype and sometimes even lack of benign or malignant designation^4–6^. This begs the question of whether tumors that are diagnosed as a low-grade cartilaginous lesion after initial percutaneous biopsy should be followed by a repeat biopsy prior to wide resection of the lesion, especially when imaging findings suggest more aggressive biology. This often requires an open biopsy which is morbid in almost any pelvic location and results in contamination of tissues that may not be otherwise contaminated.

We hypothesized that given the aggressive nature of cartilaginous lesions of the pelvis, and high potential for sampling error in these lesions, patients with the appearance of a low-grade cartilaginous tumor on initial biopsy have a high likelihood of upgrading, and/or confirming malignant designation upon histopathologic review of the entire resection specimen. This presents a unique dilemma. Since wide excision of pelvic chondrosarcomas typically requires some form of hemipelvectomy, it can be discomfiting for a surgeon to recommend an extremely morbid operation for what may appear to be a low-grade tumor. Additionally, patients may research low grade cartilage tumors and conclude that this does not require wide excision at all, given the literature about atypical cartilaginous tumors in the extremities. Our institution has not historically performed open biopsies of periacetabular cartilaginous tumors in order to reach a higher degree of diagnostic certainty. Rather, we recommend wide excision for all patients in whom the clinical and radiological features are consistent with chondrosarcoma, regardless of the grade implied by a preoperative needle biopsy. It should be noted that with these types of tumors, a multidisciplinary board (consisting of Orthopaedic oncology, Medical oncology, Radiation oncology, Radiology, and Pathology) is convened to discuss each individual case from multiple perspectives to allow for a cohesive and appropriate treatment plan. This review was motivated by a desire to answer the question, “Has this paradigm served our patients well.” In this paper, we were somewhat surprised to demonstrate a significant degree of discrepancy between preoperative biopsy and final diagnosis. However, we were pleased to note that there was no case in which the recommendation for a wide excision would have been different had the final tumor grade been known preoperatively.

Histologically, chondrosarcomas of the appendicular and axial skeleton are classified on a grading scale of 1–3 or as dedifferentiated according to their nuclear size, nuclear staining, mitotic activity, and cellularity^1,2,4^. This grading system is correlated with recurrence rates, metastasis, and prognosis^1,2,4^. The biological behavior of these tumors’ ranges from locally aggressive, yet with little capacity for metastasis (grade 1), to extremely aggressive with a greater potential to metastasize (grade 3 and dedifferentiated)^1,2,4^. As of 2013, grade 1 chondrosarcomas were reclassified by the World Health Organization as “atypical cartilaginous tumors” when found in the appendicular skeleton, given that tumors in this location were found to rarely metastasize^1,13,14^. However, the nomenclature “grade 1 chondrosarcoma” was retained for tumors found in the axial skeleton due to their locally aggressive behavior, greater potential to metastasize, and association with a poorer prognosis relative to their appendicular counterparts^1^.

This latter feature is controversial and, despite the recent change in nomenclature, there is controversy regarding the biological distinction between the low-grade cartilaginous tumors in the axial and appendicular skeletons. Some studies have noted no difference in overall survival based on tumor location^15^, while others have found that axial grade I chondrosarcomas are more aggressive and are associated with a poorer prognosis than their appendicular counterparts^16,17^. Finally, dedifferentiated chondrosarcoma represents a unique subtype of tumor with histology consisting of cartilaginous tumor and non-cartilaginous high-grade component^1,2,18^. This subtype is highly invasive and associated with distant metastasis and poor prognosis^1,2,18^.

Three main biopsy methods are available for preoperative tissue sampling: core needle biopsy, fine needle aspiration, and open biopsy. Due to the complex anatomy of the periacetabular region, open biopsy is difficult and poses significant risk of oncological contamination of nearby tissues. In these cases, percutaneous biopsy methods such as core needle biopsy and fine needle aspiration are typically utilized. Treatment of patients with chondrosarcoma varies depending on the classification and the anatomical location of the tumor. Appropriate imaging is beneficial to aid in the categorization of these tumors and serves as a critical piece of evidence for the multifaceted team of physicians to determine the most appropriate treatment. Because chondrosarcoma is generally chemoresistant and radioresistant, wide excision remains the main treatment available and it is imperative that the decision to undertake a wide excision be made correctly^1,11,12^.

Currently, it is widely accepted that atypical cartilaginous tumors of the appendicular skeleton may be treated with intralesional curettage^12,19–22^. In light of this, a similar approach for grade 1 chondrosarcoma of the axial skeleton seems appealing, due to the likelihood of better functional outcomes and lower complication rates^6^. However, there is controversy in the current literature regarding the difference in biological nature between the two and, therefore, whether grade 1 chondrosarcoma of the axial skeleton may be treated effectively with intralesional curettage^6,12,15–17,22^. Additionally, it is notable that among the eight patients in the present study with a preoperative diagnosis of “low grade cartilaginous tumor” (where even a definitive diagnosis of malignancy could not be given) that the final histopathological assessment was at least that of intermediate grade chondrosarcoma. The gold standard for management of intermediate grade, high grade, and dedifferentiated chondrosarcomas of the appendicular and axial skeleton is wide resection^12^. Wide resection of the periacetabular region is accomplished typically by a Type II hemipelvectomy with or without reconstruction. This surgery is accompanied by unique morbidity due to involvement of the weight bearing and functional hip joint and disruption of the hip abductors during exposure^23,24^. In no patient in the present study was this surgery performed and then found to be inappropriate based on final histopathology.

With evidence that atypical cartilaginous tumors of the appendicular skeleton may be treated with intralesional curettage and the current controversy as to the biological difference between atypical cartilaginous tumors of the appendicular skeleton and “grade 1 chondrosarcoma” of the pelvis^12,15–17,19,21,22^, decision making has become further complicated following a low-grade preoperative biopsy. This presents a dilemma for the treating surgeon: Proceed with a hemipelvectomy if the clinical and imaging features fit with chondrosarcoma or obtain more tissue for a definitive diagnosis in the form of an open biopsy?

Proceeding with Type II hemipelvectomy based on a presumptive diagnosis should not be taken lightly as it can result in significant functional loss, poor cosmesis, and psychological morbidity. Ideally the true grade of the tumor is determined in order to ensure the appropriate management for each patient. However, diagnostic imaging and percutaneous biopsy cannot always accurately identify tumor grade^4–6^ and our experience suggests that even patients with a low-grade cartilaginous lesion on initial biopsy are likely to ultimately be diagnosed with chondrosarcoma, of at least intermediate grade, on final pathologic evaluation of the entire tumor. This was confirmed in the present study. Open biopsy remains the gold standard for diagnosis of musculoskeletal tumors, but is as sociated with risks of oncological contamination which may complicate treatment^25–27^. Therefore, when presented with a preoperative low-grade tumor two options must be considered: Perform an open biopsy to confirm the grade, posing additional risk and morbidity to the patient, or proceed with Type II hemipelvectomy, with an inconclusive biopsy, and risk over management if the lesion truly is low-grade.

Our study was designed to bring some data and clarity to this treatment dilemma. The main objective of this study was to investigate the concordance of preoperative biopsy and surgical resection histological grade in patients with periacetabular chondrosarcoma at a single institution. As shown by our results, IGCNB demonstrated significant discrepancy between preoperative biopsy and surgical resection histological grade. Twelve cases of discrepancy demonstrated a final histopathology of a higher grade than that of pre-operative biopsy histology, while one case of IGCNB demonstrated a final histopathology of intermediate grade when the pre-operative biopsy histology had indicated a high-grade chondrosarcoma. However, all patients with cartilage seen on preoperative biopsy were ultimately diagnosed with chondrosarcoma and, thus, there were no cases of benign etiology.

A secondary objective of this study was to determine whether our institution’s protocol should be modified to include repeat biopsy following a preoperative biopsy revealing a low-grade cartilaginous lesion in an effort to confirm the true nature of the tumor prior to proceeding with wide resection. As shown, despite the discrepancy seen with IGCNB, there were no scenarios in which treatment decisions, including operative plan, would have been changed. In fact, the identification of a cartilaginous tumor, independent of its preoperative grade, in conjunction with clinical and imaging features of chondrosarcoma was justified to guide management in each of the patients in this study, as these three components provide complementary pieces of the diagnostic puzzle. Therefore, we suggest that percutaneous techniques, such as IGCNB, are sufficient for guiding treatment decisions even though discrepancies exist, particularly because pathology can play a crucial role in determining the tissue’s origin.

There are several limitations of this study. First, due to the nature of our single institutional study and the small patient cohort we were able to analyze our results may not be generalizable to the broader scope of patients with periacetabular chondrosarcoma. Additionally, this was a retrospective chart review with data gathered from clinical records that were not originally designed for research data collection. Not all periacetabular chondrosarcomas treated at our institution were able to be included due to incomplete medical records, therefore this sample may not be fully reflective of all patients with periacetabular chondrosarcoma. The data that we collected only reflected a patient population that underwent surgery, we did not include any patients that had low grade cartilage lesion on biopsy, benign-appearing imaging, and no subsequent operation. We also did not include any patients who elected to have definitive proton XRT, as we do not have the follow-up of definitive histopathology. Finally, pathologist interobserver variability may have resulted in inconsistent histopathological diagnoses. Although all patients were treated at our institution, not all of the preoperative biopsies were performed at our institution. Our pathologists typically obtain histology slides from outside institutions to confirm diagnosis, but there may be sampling error or bias introduced when reviewing outside slides. Finally, decisions regarding diagnosis and management of these patients was performed at our high-volume sarcoma center with a highly experienced multidisciplinary team. As a result, these limitations should be considered when analyzing the results and before applying them to clinical practice.

## Conclusion

In conclusion, pre-operative biopsy often results in undergrading, but it did not change treatment, and all patients received the appropriate management at our institution. The presence of cartilaginous tissue on preoperative biopsy from the periacetabular region, independent of grade, combined with correlative clinical and imaging features evaluated by experienced orthopedic oncologists at a high-volume sarcoma center, can be used to guide treatment for suspected chondrosarcoma. When the imaging and clinical features suggest a higher-grade tumor than the biopsy, the investigators of this paper treat the patient accordingly. Unlike treatment management of the upper and lower extremities, the investigators of the study treat all cartilage malignancies with a wide resection, regardless of grade, due to the aforementioned tumor undergrading.

To provide more precise and thorough management planning, further research is needed to identify more accurate, conservative diagnostic methods for tumors in this region. Additionally, further research on the biological nature of low-grade chondrosarcoma of the pelvis is needed to understand if these tumors always have malignant potential.

## Electronic supplementary material

Below is the link to the electronic supplementary material.

Supplementary Material 1

## Data Availability

Our data was generated per chart review at our institution and we can provide de-identified data if needed. n_cantor0822@email.campbell.edu can be contacted if someone would like to request the data from the study. We also attached the raw data to this submission.
